# Opportunistic *Elizabethkingia miricola* Infections in Intensive Care Unit, Spain

**DOI:** 10.3201/eid3004.231491

**Published:** 2024-04

**Authors:** Eva Soler-Iborte, Mario Rivera-Izquierdo, Carmen Valero-Ubierna

**Affiliations:** Hospital Universitario San Cecilio, Granada, Spain (E. Soler-Iborte, M. Rivera-Izquierdo, C. Valero-Ubierna);; Instituto biosanitario de Granada, Granada, (M. Rivera-Izquierdo);; Ciber de Epidemiología y Salud Pública, Madrid, Spain (M. Rivera-Izquierdo)

**Keywords:** Elizabethkingia miricola, bacteria, COVID-19, antimicrobial resistance, hospital-acquired infections, nosocomial infections, Spain

## Abstract

In 2021, we identified a cluster of *Elizabethkingia miricola* cases in an intensive care unit in Spain. Because *E. miricola* is not considered a special surveillance agent in Spain, whole-genome sequencing was not performed. The bacterial source was not identified. All *Elizabethkingia* species should be listed as special surveillance bacteria.

The *Elizabethkingia* genus is formed by a group of gram-negative, aerobic, and nonfermenting bacteria widely distributed in nature and environments, such as water and hospital taps (*1*). In 2003, a new a bacterial species was identified in the condensation water obtained from the Mir space station in 1997 and was assigned as *Chryseobacterium miricola* (*2*). That new species was later transferred to the *Elizabethkingia* genus and renamed *Elizabethkingia miricola* (*3*). This species is considered as an uncommon low-pathogenic agent in clinical samples, acting as an opportunistic pathogen, but since 2008, it has become an emerging bacterium of increasing relevance (*4*). *E. miricola* has not been fully epidemiologically characterized but is considered intrinsically resistant to multiple drugs (*5*).

We describe a cluster of *E. miricola* in the intensive care unit (ICU) of the Hospital Universitario San Cecilio in Granada, Spain. The index case corresponded to a 66-year-old man hospitalized for COVID-19. The microbiology service identified *E. miricola* isolates from a bronchial aspirate sample by using matrix-assisted laser desorption/ionization time-of-flight mass spectrometry and informed the ICU of a positive result on March 19, 2021. Two days later, a 70-year-old man hospitalized in the same unit for COVID-19 also tested positive for *E. miricola* in a bronchial aspirate sample sent for a previous diagnosis with tracheobronchitis associated with *Stenotrophomonas maltophilia.*

Throughout 2021, we found 13 more cases of *E. miricola* in the same ICU. Given the identification of the same species, and for clinical and epidemiologic criteria, the outbreak was considered nosocomial. However, although isolates were retained for potential future research, no whole-genome sequencing could be performed because the species is not included as special surveillance agent for the Andalusian Health System because of resource limitations. The reason for admission in 11 (73.3%) patients was COVID-19. Of the 15 case-patients, 11 (73.3%) were men and 4 (26.7%) women, 6 (40.0%) had tracheobronchitis diagnoses, 3 (20.0%) had ventilator-associated pneumonia, and 1 (6.7%) had catheter-related bacteremia; 8 (53.3%) patients died (Table).

**Table:** Characteristics of case-patients in an outbreak opportunistic *Elizabethkingia miricola* infections in an intensive care unit, Spain*

Age, y/sex	Isolation sample	ICU stay, d	Diagnosis†	Diagnosis date, 2021	Death	Reason for admission	Antimicrobial drug resistance‡
66/M	BAS	40	Isolation	Mar 17	N	COVID-19	Carbapenems, ceftazidime, cefepime, aztreonam
70/M	BAS	63	Isolation	Mar 19	N	COVID-19	Carbapenems, ceftazidime, cefepime, aztreonam
46/F	BAS	12	Isolation	Mar 27	Y	Carcinosis	Carbapenems, ceftazidime, cefepime, aztreonam, aminoglycosides
64/M	BAS	61	Tracheobronchitis	Apr 22	N	COVID-19	Carbapenems, ceftazidime, cefepime, aztreonam, piperacillin/tazobactam
57/F	BAS, catheter	20	Bacteriemia	Apr 27	Y	COVID-19	Carbapenems, ceftazidime, cefepime, aztreonam, piperacillin/tazobactam, aminoglycosides, trimethoprim/ sulfamethoxazole
50/M	BAS	58	Tracheobronchitis	May 19	Y	COVID-19	Carbapenems, ceftazidime, amikacin
52/M	BAS	37	Tracheobronchitis	May 20	N	COVID-19	Carbapenems, ceftazidime, amikacin
56/M	BAS	43	Ventilator-associated pneumonia	May 22	Y	COVID-19	Amikacin
58/F	BAS	80	Tracheobronchitis	May 29	N	COVID-19	Amikacin
58/M	BAS	30	Isolation	Jul 7	Y	Fever	Piperacillin/tazobactam, linezolid
37/F	BAS	51	Isolation	Aug 20	Y	COVID-19	Piperacillin/tazobactam, linezolid
32/M	BAS	93	Tracheobronchitis	Sep 18	Y	COVID-19	Piperacillin/tazobactam, linezolid
74/M	BAS	27	Tracheobronchitis	Sep 28	Y	Epileptic seizures	Piperacillin/tazobactam, linezolid
61/M	BAS	27	Ventilator-associated pneumonia	Oct 27	N	Septic shock	Piperacillin/tazobactam, linezolid, vancomycin,
73/M	BAS	15	Ventilator-associated pneumonia	Dec 30	N	COVID-19	Piperacillin/tazobactam, linezolid, levofloxacin

*BAS, bronchoaspiration; ICU, intensive care unit. †Isolation means that no other clinical pathology was reported by the responsible physician in the ICU. Therefore, *E. miricola* identification was considered as asymptomatic colonization. ‡Antibiograms were compatible with the same agent, but development of new resistances was identified during the 9-mo outbreak. Trimethoprim/sulfamethoxazole showed the best antibiogram sensitivity: 10 patients showed sensitivity, 4 intermediate sensitivity, and only 1 showed resistance. Sensitivity to levofloxacin was observed for 7 (46.7%) patients; 4 more showed intermediate sensitivity.

All patients received steroid treatment during their ICU stays. All case-patients were intubated during their hospitalization. The average length of ICU stay was 43.8 days, and the length between admission and identification of the agent was long, a mean of 26.4 days.

Because 290 days elapsed from identification of the index case (March 19) to identification of the last case (December 30), we assumed persistence of the agent in the ICU environment. Nevertheless, despite a search of environmental and surface samples, the definitive focus of persistence was not identified. Because of possible cross-transmission in a unit with such vulnerable patients, we notified the Service of Preventive Medicine and Public Health, which initiated prevention measures. Because of the lack of available knowledge related to *E. miricola* and closely related species, we reinforced standard precautions and established contact precautions. Finally, by December 2021, we conducted a thorough disinfection of all surfaces in the ICU, after which no more cases were identified.

In other countries, cases of multidrug resistance were identified in the context of antimicrobial drug pressure and cases of sepsis and pneumonia were diagnosed among immunosuppressed patients (*5*). In our hospital, 8 (53.3%) patients died. The average time from bacterial isolation to death was 18.2 (range 2–65) days.

*Elisabethkingia* isolates are usually resistant to multiple antibiotics. In analyses of different isolates collected in South Korea and Taiwan (*5*), all *E. miricola* isolates were resistant to cephalosporins, aminoglycosides, and carbapenems. Those data are similar to results obtained in our hospital (Table). A study conducted in Switzerland found genes encoding metallo-β-lactamases in a multidrug-resistant *E. miricola* isolated from the urine of a 2-year-old boy (*6*). Those genes provide resistance to penicillin-β-lactamase inhibitor combinations, carbapenems, cefotaxime, and cefoxitin. Trimethoprim/sulfamethoxazole showed the best antibiogram sensitivity in our outbreak, only 1 of 15 patients showed resistance (Table).

In summary, our study underlines the need to find *Elizabethkingia* spp. bacteria in ICUs. In addition, all species in the *Elizabethkingia* genus should be listed as special surveillance bacteria due to their capacity to cause major illness and death in vulnerable patients. Future studies analyzing differences in the outcomes between patients with *E. miricola* and other patients admitted to ICU, including patient characteristics and treatments, could expand on the information provided in this study. Finally, to enable early detection of outbreaks of intrinsically antimicrobial-resistant bacteria, modify patient treatment, and save lives, whole-genome sequencing needs to be instituted when rare agents not previously considered for special surveillance are identified.

## References

[1] Zdziarski P, Paściak M, Rogala K, Korzeniowska-Kowal A, Gamian A. *Elizabethkingia miricola* as an opportunistic oral pathogen associated with superinfectious complications in humoral immunodeficiency: a case report. BMC Infect Dis. 2017;17:763. 10.1186/s12879-017-2886-7 29233117 PMC5727958

[2] Li Y, Kawamura Y, Fujiwara N, Naka T, Liu H, Huang X, et al. *Chryseobacterium miricola* sp. nov., a novel species isolated from condensation water of space station Mir. Syst Appl Microbiol. 2003;26:523–8. 10.1078/072320203770865828 14666980

[3] Kim KK, Kim MK, Lim JH, Park HY, Lee ST. Transfer of *Chryseobacterium meningosepticum* and *Chryseobacterium miricola* to *Elizabethkingia* gen. nov. as *Elizabethkingia meningoseptica* comb. nov. and *Elizabethkingia miricola* comb. nov. Int J Syst Evol Microbiol. 2005;55:1287–93. 10.1099/ijs.0.63541-0 15879269

[4] Gupta P, Zaman K, Mohan B, Taneja N. *Elizabethkingia miricola*: A rare non-fermenter causing urinary tract infection. World J Clin Cases. 2017;5:187–90. 10.12998/wjcc.v5.i5.187 28560237 PMC5434319

[5] Lin JN, Lai CH, Yang CH, Huang YH. *Elizabethkingia* infections in humans: from genomics to clinics. Microorganisms. 2019;7:295. 10.3390/microorganisms7090295 31466280 PMC6780780

[6] Colapietro M, Endimiani A, Sabatini A, Marcoccia F, Celenza G, Segatore B, et al. BlaB-15, a new BlaB metallo-β-lactamase variant found in an *Elizabethkingia miricola* clinical isolate. Diagn Microbiol Infect Dis. 2016;85:195–7. 10.1016/j.diagmicrobio.2015.11.016 27033632

